# Structure elucidation and quantification of the reduction products of anticancer Pt(iv) prodrugs by electrochemistry/mass spectrometry (EC-MS)†
†Electronic supplementary information (ESI) available: Experimental part, mass voltammograms, EC-LC-MS analysis. See DOI: 10.1039/c8an00258d


**DOI:** 10.1039/c8an00258d

**Published:** 2018-04-09

**Authors:** L. M. Frensemeier, J. Mayr, G. Koellensperger, B. K. Keppler, C. R. Kowol, U. Karst

**Affiliations:** a Institute of Inorganic and Analytical Chemistry , University of Münster , Corrensstr. 28/30 , 48149 Münster , Germany . Email: christian.kowol@univie.ac.at ; Email: uk@uni-muenster.de; b Institute of Inorganic Chemistry , Faculty of Chemistry , University of Vienna , Währinger Str. 42 , 1090 Vienna , Austria; c Institute of Analytical Chemistry , Faculty of Chemistry , University of Vienna , Währinger Str. 38 , 1090 Vienna , Austria

## Abstract

Pt(iv) prodrugs are a class of promising anticancer agents, which are activated by reduction to the active Pt(ii) species. Consequently, the reduction process is a crucial parameter. Herein, a new approach using electrochemistry (EC) coupled to liquid chromatography (LC) and electrospray ionization-mass spectrometry (ESI-MS) or inductively coupled plasma (ICP)-MS was applied. This enabled getting insights into the differences in the reduction and ligand release of platinum(iv) complexes with varying equatorial core structures.

## Abstract 2

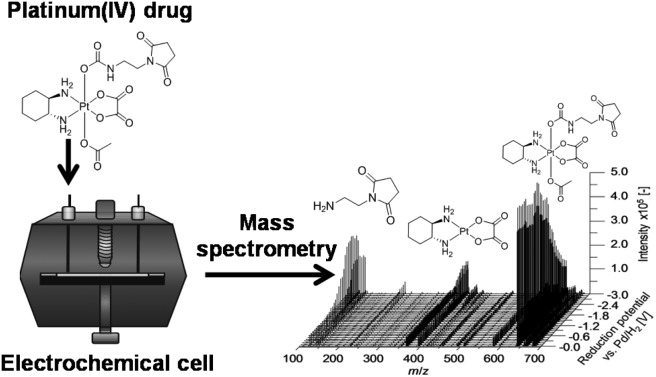
A novel analytical approach for the identification/quantification of the reduction products of platinum(iv) complexes is presented.

## 

Platinum-containing anticancer drugs represent one of the main classes of chemotherapeutics^1^–^3^ and the Pt(ii) derivatives cisplatin, carboplatin and oxaliplatin are clinically approved worldwide. Despite their success in the treatment of diverse types of cancer, severe side effects are frequently associated with this kind of therapy.^1^,^4^,^5^ Consequently, current research focuses on the development of alternative Pt compounds entailing less adverse effects. The design and application of Pt(iv) prodrugs is one main approach that aims at achieving this goal.^6^,^7^ In contrast to the Pt(ii) complexes, Pt(iv) compounds bear two additional axial ligands and are kinetically more stable. Moreover, the introduction of appropriate axial targeting ligands enables a more tumor-selective drug design.^6^–^11^ Reduction of the prodrugs favorably occurs in the reductive milieu of the tumor tissue with formation of the active Pt(ii) drug.^2^,^3^,^12^–^17^ Thereby, the reduction efficiency is dependent on both the equatorial and axial ligands.^2^,^6^,^18^ Conventionally, the reduction behavior is studied with a reducing agent such as ascorbate in buffered solution or *via* cyclic voltammetry (CV).^18^–^21^ In the case of CV, usually solely the redox potentials are estimated without any structural information about the formed products. Using reductants and subsequent mass spectrometric (MS) or nuclear magnetic resonance (NMR) analysis may allow for the identification of at least the main product(s). However, this method is often only successful after isotopic labeling of the ligands and additionally solely for Pt(iv) complexes possessing a cisplatin-like equatorial core structure, since in the case of oxaliplatin- or carboplatin-based Pt(iv) derivatives, the reduction rate with ascorbate is very slow.^19^,^22^

In the present study, an alternative, purely instrumental, fast approach for the simulation of reduction reactions of Pt(iv) prodrugs is introduced. This approach comprises an electrochemical cell coupled online to liquid chromatography (LC) and MS for species characterization (Fig. 1). The set-up is well known for its powerful potential in terms of metabolism simulation under oxidative conditions.^23^–^27^ However, up to now, the application of EC for studying the reductive transformation has only rarely been investigated and only few examples exist in the literature.^28^–^31^ The EC reduction and subsequent MS analysis of metal-based small molecules is a novel, fast and easy tool to determine the reduction products, which are also likely to occur in a biological system.

**Fig. 1:**
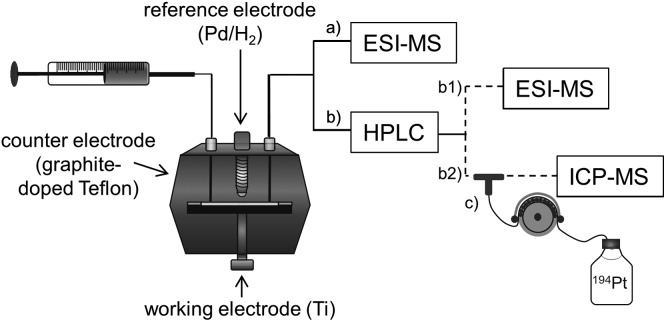
Instrumental setup for the electrochemical reduction of Pt(iv) compounds. (a) Species identification by EC-ESI-MS; (b1) EC-LC-ESI-MS or (b2) EC-LC-ICP-MS analysis of Pt(iv) compounds; (c) post-column isotopic dilution analysis for the quantification of Pt species.

Recently, Mayr *et al.* synthesized the first mono-maleimide-functionalized oxaliplatin- and cisplatin-based Pt(iv) complexes, which showed an outstanding antitumor activity in CT-26-bearing mice *in vivo*.^19^ Notably, the oxaliplatin derivatives were strongly superior compared to a cisplatin complex.

Consequently, in the present work, the reduction of two oxaliplatin and one cisplatin-based Pt(iv) compound was investigated (the stable succinimide derivatives were used instead of the maleimide complexes to avoid hydrolysis) and compared to satraplatin (Fig. 2), which has already been tested in clinical phase III studies. Electrospray (ESI)-MS analysis of the electrochemically reduced Pt(iv) derivatives allowed product identification. The implementation of a LC separation between the EC cell and ESI-MS or inductively coupled plasma (ICP)-MS for detection enabled a characterization of both the main reduction product and the by-products that are formed. The different Pt species were quantified using isotopic dilution analysis (IDA), thus providing a tool for the estimation of the influence of the structure of the Pt(iv) complexes on their reduction behavior and the formation of (by-)products.

**Fig. 2:**
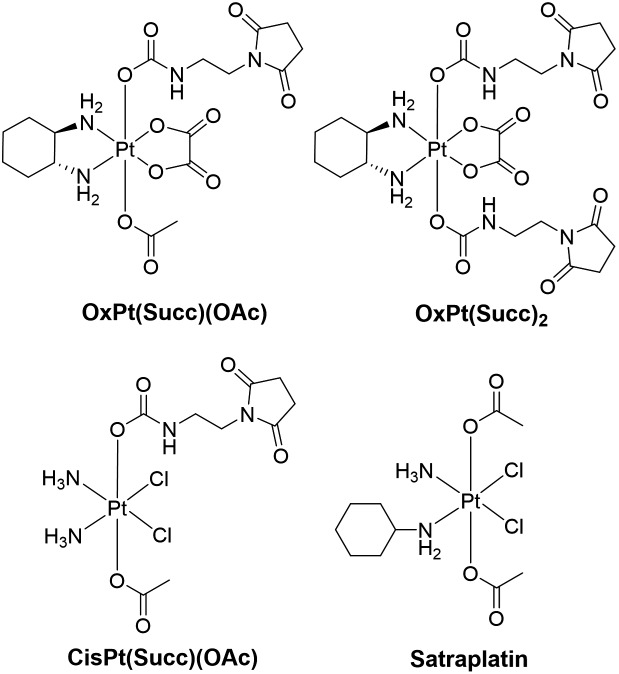
Chemical structures of the investigated Pt(iv) compounds.

In a first experiment, the reduction of the different Pt(iv) compounds was investigated by coupling the EC cell to an Orbitrap-based ESI-MS (Exactive). A potential ramp from 0.0 to –3.0 V (*vs.* Pd/H_2_) was applied to the cell and the effluent was subsequently analyzed. By plotting the obtained mass spectra in dependency of the applied potential, a mass voltammogram (MV) (exemplarily shown for OxPt(Succ)(OAc) in Fig. 3) can be generated. OxPt(Succ)(OAc) was identified with a mass-to-charge ratio (*m*/*z*) of 642.1370 (Δ*m* 0.2 ppm). The detected isotopic pattern resulting from the natural distribution is in good accordance with the theoretical one. From the MV, the reduction of OxPt(Succ)(OAc) can easily be identified by the decreasing signal intensity at decreasing potential. Simultaneously, increasing signals for oxaliplatin (*m*/*z* 398.0676, Δ*m* 0.3 ppm) and the axial succinimide ligand (Succ, *m*/*z* 143.0815 after loss of CO_2_ during ESI ionization, Δ*m* < 0.05 ppm) were detected. The highest conversion to oxaliplatin was observed at –3.0 V. Therefore, the potential was set to –3.0 V for the following experiments.

**Fig. 3:**
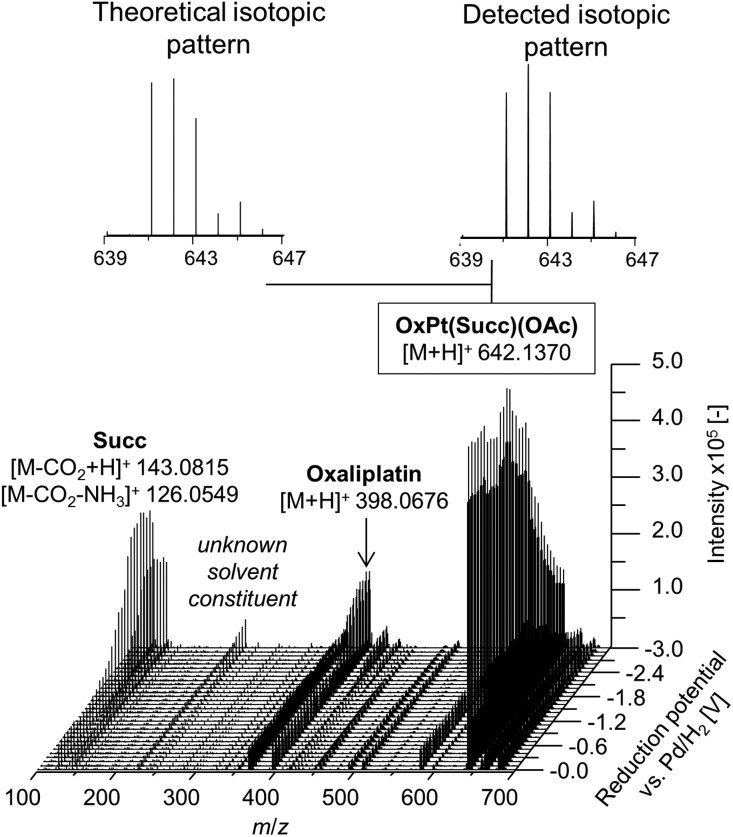
Mass voltammogram of OxPt(Succ)(OAc). A potential ramp from 0.0 to –3.0 V (*vs.* Pd/H_2_) was applied to the electrochemical cell.

The MVs of the other three investigated compounds coincide closely with that of OxPt(Succ)(OAc) and are provided in the ESI (Fig. S1†). A maximum conversion to the expected reduction products (oxaliplatin, cisplatin and *cis*-amminedichlorido(cyclohexylamine)platinum(ii) ([PtCl_2_(cyclohexylamine)(NH_3_)])) was obtained at a potential of –3.0 V. Notably, the EC reduction did not lead to by-product formation detectable by direct ESI-MS analysis. These findings may result from an insufficient sensitivity of the ESI-MS detection for products formed in low concentrations.

In order to characterize the EC conversion in more detail and to identify potential by-products more effectively, a LC separation was added between the EC cell and the MS. Additionally, ICP-MS instead of the ESI-MS was used for analysis of the formed (by-)products. The Pt(iv) compounds were reduced at a constant potential of –3.0 V and the cell effluent was analyzed by online LC-ESI-MS and LC-ICP-MS. Moreover, quantification of the Pt species was performed using IDA, which allowed for an estimation of the influence of the structure of the Pt(iv) prodrugs on their reduction behavior.

The obtained chromatograms are shown in Fig. 4 and Fig. S2† and the respective quantification results are summarized in Fig. 5.

**Fig. 4:**
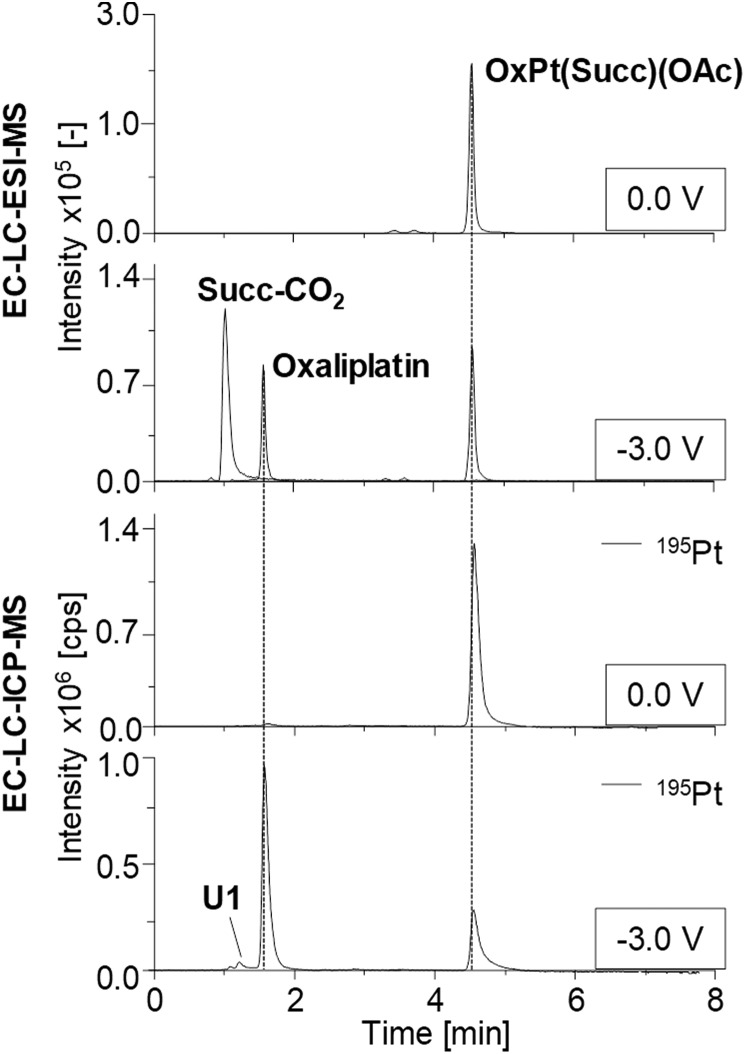
Chromatographic separation of OxPt(Succ)(OAc) and its respective reduction products obtained by electrochemical conversion at a constant potential of –3.0 V *vs.* Pd/H_2_. Detection was performed using ESI-MS and complementary ICP-MS.

**Fig. 5:**
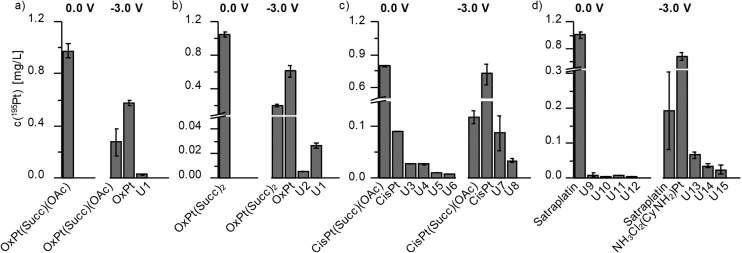
Quantification results for the different Pt species with (–3.0 V) and without (0.0 V) EC reduction based on a post-column isotopic dilution analysis.

Detected species as well as their retention times (*t*_R_), the determined concentration and the corresponding relative standard deviations obtained from triplicate measurements are summarized in Table S5.† The *t*_R_ of all Pt(iv) prodrugs and their expected reduction products oxaliplatin, cisplatin and [PtCl_2_(cyclohexylamine)(NH_3_)] were found to coincide between the LC-ESI-MS and LC-ICP-MS analysis.

Investigations on OxPt(Succ)(OAc) and OxPt(Succ)_2_ (Fig. 4, S2a† and Fig. 5a/b) revealed single signals for the complexes themselves at 0.0 V by both ESI-MS and ICP-MS. After reduction, additional peaks for oxaliplatin and the succinimide ligand after loss of CO_2_ (Succ-CO_2_) were observed using ESI-MS detection. The ICP-MS chromatogram revealed intensive peaks of oxaliplatin, which represented 64% of all Pt species detected in case of OxPt(Succ)(OAc) and 73% in case of OxPt(Succ)_2_. Furthermore, two unknown Pt compounds, U1 for both complexes and U2 for OxPt(Succ)_2_, were observed, which could not be detected by ESI-MS, presumably due to a concentration below the limit of detection (LOD; this applies also to the following species denoted with U).

The LC-ESI-MS analysis of CisPt(Succ)(OAc) (Fig. S2b†) elucidated a small amount of Succ-CO_2_ already at 0.0 V, thus indicating a partial degradation of the parent compound in solution. These findings could be confirmed with ICP-MS detection at 0.0 V. Not only a signal of CisPt(Succ)(OAc) was observed, but also several other Pt species (*t*_R_ = 0.7 min (CisPt, 90.5 μg L^–1^), 1.0 min (U3, 27.7 μg L^–1^), 1.2 min (U4, 27.0 μg L^–1^), 1.5 min (U5, 10.9 μg L^–1^) and 3.1 min (U6, 6.94 μg L^–1^)) (Fig. 5c). Notably, these results were unexpected due to the generally high stability of Pt(iv) compounds and therefore are assumed to be associated with the applied solvent for electrolysis. Analysis of CisPt(Succ)(OAc) after EC reduction showed a signal corresponding to cisplatin with a concentration of 580 μg L^–1^ Pt, representing 71% of the total Pt species. This cisplatin peak was also confirmed with ESI-MS, together with Succ-CO_2_. In the ICP-MS chromatogram, however, two additional signals were detected corresponding to the by-products U7 and U8 (*c*(^195^Pt) = 87.2 and 33.6 μg L^–1^). The results for the investigations on satraplatin are shown in Fig. S2c† and Fig. 5d. With ESI-MS, only satraplatin was detected at 0.0 V. The ICP-MS measurement showed one major signal for ^195^Pt at the same *t*_R_. In addition, some very low abundant Pt species (U9–U12) with *c*(^195^Pt) < 10 μg L^–1^ (which together is only ∼2% of total Pt) were observed. After EC reduction, [PtCl_2_(NH_3_)(NH_2_Cl_2_)] was identified as the main reduction product (*c*(^195^Pt) = 573 μg L^–1^, 64% of total Pt). In addition, by-products at *t*_R_ = 0.9 min (U13, 67.7 μg L^–1^), 5.4 min (U14, 34.9 μg L^–1^), 5.7 min (U15, 24.7 μg L^–1^) were found by means of ICP-MS analysis.

Comparing the investigated Pt(iv) complexes, the EC reduction at –3.0 V resulted in conversions to the expected Pt(ii) species in high yields of 64–73% of total Pt content. These results indicate that the structure of the Pt(iv) complexes, regarding both axial and equatorial ligands, have no significant influence on the reduction yield under the applied EC conditions. Most likely, this is due to small differences in the reduction potential. According to the literature, equatorial ligands generally less impact the reduction potential compared to axial ligands,^2^,^6^,^18^ which however were quite similar in the investigated complexes. Thus, the impact of acetate compared to the carbamate-coordinated Succ ligand seems insufficient to strongly influence the reduction potentials and efficiencies. To confirm this assumption, the reduction behavior of OxPt(OH)_2_ was studied, which is known to possess a much lower reduction potential due to the two stronger electron-donating hydroxido ligands.^32^ The corresponding MV is presented in Fig. S3.† By means of EC, no reduction was observed down to –3.0 V, which supports the lower redox potential compared to the other investigated compounds and proves that this method is also able to detect differences in the redox potential of Pt(iv) complexes. Comparing OxPt(Succ)(OAc) and OxPt(Succ)_2_ to CisPt(Succ)(OAc) and satraplatin, a more selective reduction for oxaliplatin was observed. Thus, the amount of by-product formation (% of Pt amount of the products) for the oxaliplatin derivatives was only ∼5%, whereas for the cisplatin derivative and satraplatin 17–18% by-product generation was obtained. This can most likely be explained by the two equatorial chelate ligands in case of oxaliplatin compared to the mono-dentate am(m)ine and chlorido ligands in case of the cisplatin derivative and satraplatin. EC reduction of OxPt(Succ)(OAc) and OxPt(Succ)_2_ lead to a decrease of the educt concentrations of 72% and 81%, respectively. In comparison, 81% of satraplatin and 85% of CisPt(Succ)(OAc) were converted. Thus, the cisplatin complex showed the highest conversion rate, which, however, did not result in the highest product formation but in increased by-product formation (∼17%).

Generally, the formation of other complexes than the simple equatorial Pt(ii) species during Pt(iv) reduction is a known phenomenon^2^ and was, *e.g.*, in detail studied with isotopically labeled satraplatin.^33^ Although the exact characterization of the by-products by ESI-MS was not possible due to concentrations below the LOD, the differences observed in the formation of by-products during EC reduction gave a good estimate for the stability and selectivity of product formation. Based on these findings, it can be expected that EC reduction is a valuable tool for the prediction of these important parameters and the main reduction products for Pt(iv) complexes in general. Although in theory, the reduction could be also studied using chemical reducing agents, the reduction processes of, *e.g.*, oxaliplatin(iv) derivatives like OxPt(Succ)(OAc) or OxPt(Succ)_2_ is too slow to obtain sufficient amounts of the reduced species (<10% after 24 h using 10 eq. of ascorbic acid).^19^ The same holds true for Pt(iv) complexes with equatorial core structures bearing a dicarboxylate ligand, like in case of carboplatin-based Pt(iv) complexes. Therefore, to date, only the reduction processes of complexes with a cisplatin, satraplatin or other dichlorido core structure could be investigated.^2^

## Conclusions

Taken together, the here presented EC-LC-MS technology has proven to serve as the first method which allows the fast investigation of the reduction behavior of Pt(iv) complexes as well as the product identification and quantification. Further studies will show the suitability of this method also for other bioactive metal complexes, which are activated by reduction such as Ru(iii) or Co(iii) compounds.

## Conflicts of interest

There are no conflicts to declare.

## Supplementary Material

Supplementary information
