# Supplementary material for: Structure elucidation and quantification of the reduction products of anticancer Pt(iv) prodrugs by electrochemistry/mass spectrometry (EC-MS)
Source: Analyst. 2018 Apr 9;143(9):1997–2001. doi: 10.1039/c8an00258d (PMC5933000; doi:10.1039/c8an00258d)
Supplement: Supplementary file 1 [file AN-143-C8AN00258D-s001.pdf]

This journal is © The Royal Society of Chemistry 2018

Electronic Supporting Information

Structure elucidation and quantification of the reduction products of anticancer Pt(IV) prodrugs by electrochemistry/mass spectrometry (EC-MS)

L. M. Frensemeier[1], J. Mayr[2], G. Koellensperger[3], B. K. Keppler[2], C. R. Kowol*[2] and U. Karst*[1]

[1] Institute of Inorganic and Analytical Chemistry, University of Münster, Corrensstr. 28/30, 48149 Münster, Germany
[2] Institute of Inorganic Chemistry, Faculty of Chemistry, University of Vienna, Währinger Str. 42, 1090 Vienna, Austria
[3] Institute of Analytical Chemistry, Faculty of Chemistry, University of Vienna, Währinger Str. 38,1090 Vienna, Austria

*Corresponding authors:

Prof. Dr. Uwe Karst, Institute of Inorganic and Analytical Chemistry, University of Münster, Corrensstraße 30, 48149 Münster, Germany, Phone: +49-251-8333141, Fax: +49-251-8336013, E-mail: uk@uni-muenster.de

Ass.-Prof. Dr. Christian Kowol, University of Vienna, Institute of Inorganic Chemistry, Faculty of Chemistry, University of Vienna, Währinger Str. 42, 1090 Vienna, Austria, Phone: +43-1-4277-52609, E-mail: christian.kowol@univie.ac.at

Reagents. The Pt(IV) compounds OxPt(Succ)(OAc), $OxPt(Succ)_2$, CisPt(Succ)(OAc) (Succ = 2-(2,5-dioxopyrrolidin-1-yl)ethylcarbamato) were synthesized at the Institute of Inorganic Chemistry, University of Vienna according to protocols published by Pichler *et al.* and Mayr *et al.*[19,34] Satraplatin was purchased from Boc Science (New York, USA). Ammonium formate (trace metal basis) was obtained from Sigma Aldrich (Steinheim, Germany). Acetonitrile (hypergrade for LC-MS), nitric acid (suprapure, 65%) and the platinum ICP standard (1 g/L) were purchased from Merck (Darmstadt, Germany). The platinum standard solution enriched with ^{194}Pt (0.02% ^{192}Pt, 97.08% ^{194}Pt, 2.04% ^{195}Pt, 0.72% ^{196}Pt, and 0.14% ^{198}Pt) was diluted from base material from Isoflex (San Francisco, USA). Water for sample preparation and HPLC was purified using an Aquatron A4000D system (Barloworld Scientific, Nemour, France). Prior to application, all solutions were degassed using ultrasonication.

Electrochemical Reduction. Electrochemical reduction was performed using an electrochemical thin-layer cell (FlexCell, Antec Scientific, Zoeterwoude, The Netherlands) equipped with titanium as cathode material. The anode consisted of graphite-doped Teflon and for potential stabilization a reference electrode made of Pd/H_2 was used. A potential ramp (10 mV/s) from 0 to -3.0 V was applied to the cell to record mass voltammograms. Pt(IV) solutions (10 µmol/L) were prepared in solutions of 10 mmol/L ammonium formate (NH_4FA; adjusted to pH 7.4 using ammonia) and acetonitrile (ACN) (50,50, *v*/*v*) and were continuously pumped through the cell using a flow rate of 10 µL/min delivered by a syringe pump (Model 74900, Cole Parmer, Vernon Hills, IL, USA). The cell effluent was subsequently introduced into an Orbitrap-based high-resolution MS (Exactive, Thermo Fisher Scientific, Bremen, Germany) operated in the positive electrospray ionization mode. Detailed MS parameters are listed in Table S1. Data processing was carried out using the software XCalibur 2.1 (Thermo Fisher Scientific) and OriginPro 2016 (OriginLab, Northhampton, MA, USA).

Table S1. Mass spectrometric parameters of the Exactive Orbitrap-MS for the generation of mass voltammograms of the Pt(IV) compounds.

parameter	OxPt(Succ)(OAc)	OxPt(Succ)$_2$	CisPt(Succ)(OAc)	Satraplatin
ionization mode	ESI(+)	ESI(+)	ESI(+)	ESI(+)
scan range [*m*/*z*]	100-1000	100-1000	100-1000	100-1200
resolution	high (50,000 @ 2Hz)	high (50,000 @ 2Hz)	high (50,000 @ 2Hz)	high (50,000 @ 2Hz)
polarity	positive	positive	positive	positive
microscans	1	1	1	1
lock masses	off	off	off	off
automatic gain control (AGC) target	balanced	balanced	balanced	balanced
maximum injection time [ms]	50.0	50.0	50.0	50.0
sheath-gas flow rate [a.u.]	10.0	10.0	10.0	10.0
aux-gas flow rate [a.u.]	0.00	0.00	0.00	0.00
sweep-gas flow rate [a.u.]	0.00	0.00	0.00	0.00
spray voltage [kV]	4.00	4.00	4.00	3.50
capillary temperature [°C]	300	300	250	250
capillary voltage [V]	50.0	55.0	250	30.0
tube lens voltage [V]	105.0	120.0	85.0	85.0
skimmer voltage [V]	26.0	24.0	18.0	18.0

Chromatographic separation. Separation of Pt(IV) prodrugs and their reduction products was performed by implementing an HPLC system (Shimadzu, Kyoto, Japan) between the electrochemical cell and an ESI-MS (Exactive) or an ICP-MS (iCAP Qc, Thermo Fisher Scientific, Bremen, Germany), respectively. The HPLC system comprised two LC-10ADVP pumps, a SIL-10A autosampler, a SCL-10AVP system controller, a DGC-14A degasser and a CTO-10ASVP column oven. System control was performed using the software LCSolution 1.2.2 (Shimadzu). Detailed MS parameters are listed in Tables S2 and S3. Reduction of the Pt(IV) compounds (10 µmol/L for LC-ESI-MS analysis, and 5 µmol/L for LC-ICP-MS analysis) was carried out at a constant potential of -3.0 V. The effluent of the cell was collected in an injection loop of a six-port valve (Rheodyne IDEX Health Science, Oak Habor, WA, USA) and transferred onto a polar-embedded C18-based column (ProntoSIL C18 ace-EPS, 100 x 2.0 mm, 3 µm, Bischoff Chromatography, Leonberg, Germany) by switching the valve. Gradient elution was applied using the gradient profile shown in

Table S4 and ammonium formate (10 mM, pH 7.4) as eluent A and acetonitrile as eluent B at a flow rate of 300 µL/min.

Table S2. Mass spectrometric parameters of the Exactive Orbitrap-MS for the EC-LC-ESI-MS analysis of the Pt(IV) species.

Parameters	OxPt(Succ)(OAc)	OxPt(Succ)$_2$	CisPt(Succ)(OAc)	Satraplatin
ionization mode	ESI(+)	ESI(+)	ESI(+)	ESI(+)
scan range [*m*/*z*]	100-1200	100-1500	100-1200	100-1200
resolution	high (50,000 @ 2Hz)	high (50,000 @ 2Hz)	high (50,000 @ 2Hz)	high (50,000 @ 2Hz)
polarity	positive	positive	positive	positive
microscans	1	1	1	1
lock masses	off	off	off	off
AGC target	balanced	balanced	balanced	balanced
maximum injection time [ms]	100.0	100.0	100.0	250.0
sheath-gas flow rate	60.00	60.0	60.0	60.0
aux-gas flow rate	10.0	10.0	10.0	10.0
sweep-gas flow rate	0.00	0.00	0.00	0.00
spray voltage [kV]	4.00	4.00	3.50	3.50
capillary temperature [°C]	350	350	350	350
capillary voltage [V]	50.0	67.5	25.0	30.0
tube lens voltage [V]	120.0	140.0	65.0	85.0
skimmer voltage [V]	18.0	20.0	20.0	18.0

Table S3. Mass spectrometric parameters of the iCAP Qc for EC-LC-(IDA-)ICP-MS analysis of the Pt(IV) compounds

parameter	value
spray chamber geometry	cyclonic (quartz)
spray chamber temperature [°C]	-3
nebulizer	PFA MicroFlow ST
injector	1 mm
sampler	Pt
skimmer	Pt
power [W]	1550
cool gas flow [L/min]	14.0
auxiliary gas flow [L/min]	0.8
nebulizer gas flow [L/min]	0.6

additional gas	O_2 (5% (v/v))
extraction lens voltage [V]	-204.3
detector voltage [V]	1787
dwell time [s]	0.05 (^{194}Pt, ^{195}Pt, ^{196}Pt, ^{198}Pt)

Table S4. LC elution profile for the separation of a) OxPt(Succ)(OAc) and $OxPt(Succ)_2$, b) CisPt(Succ)(OAc) and c) Satraplatin. The mobile phase comprised NH_4FA (10 mM, pH 7.4) (eluent A) and ACN (eluent B) at a flow rate of 300 µL/min.

a)

t [min]	2	8	10	11	18
eluent B [%]	5	40	40	5	5

b) isocratic elution at 5% B over 5 min

c)

t [min]	2	9	12	12.5	17
eluent B [%]	5	60	60	5	5

Quantification by means of EC-LC-IDA-ICP-MS. Species unspecific quantification was carried out by means of isotope dilution analysis (IDA). Therefore, a ^{194}Pt enriched solution (20 µg/L) in aqueous HNO_3 (2%, *v*/*v*) was continuously added in a post-column setup *via* a T-connection using a peristaltic pump delivering a flow rate of 170 µL/min. Prior to analysis, the detector dead time of the electron multiplier was determined based on a platinum ICP standard (0.5-30 µg/L) and corrected using QTegra software (Thermo Fisher Scientific). Mass-bias correction was performed by measuring five injections of diluted Pt ICP standard (10 µg/L). Analysis of the ICP standard and ^{194}Pt-enriched standard as well as an equimolar mixture of both (five injections per sample) enabled for reversed isotope dilution and validation of the developed method.

The obtained ICP-MS chromatograms (in cps) were converted into mass-flow chromatograms (in ng/min) using the isotope dilution equation introduced by Rodríguez-González *et al.*[35] applying the exponential model for mass-bias correction. Integration of the peaks using OriginPro 2016 (OriginLab) and correlation to the injection volume yielded the platinum amount of the species.

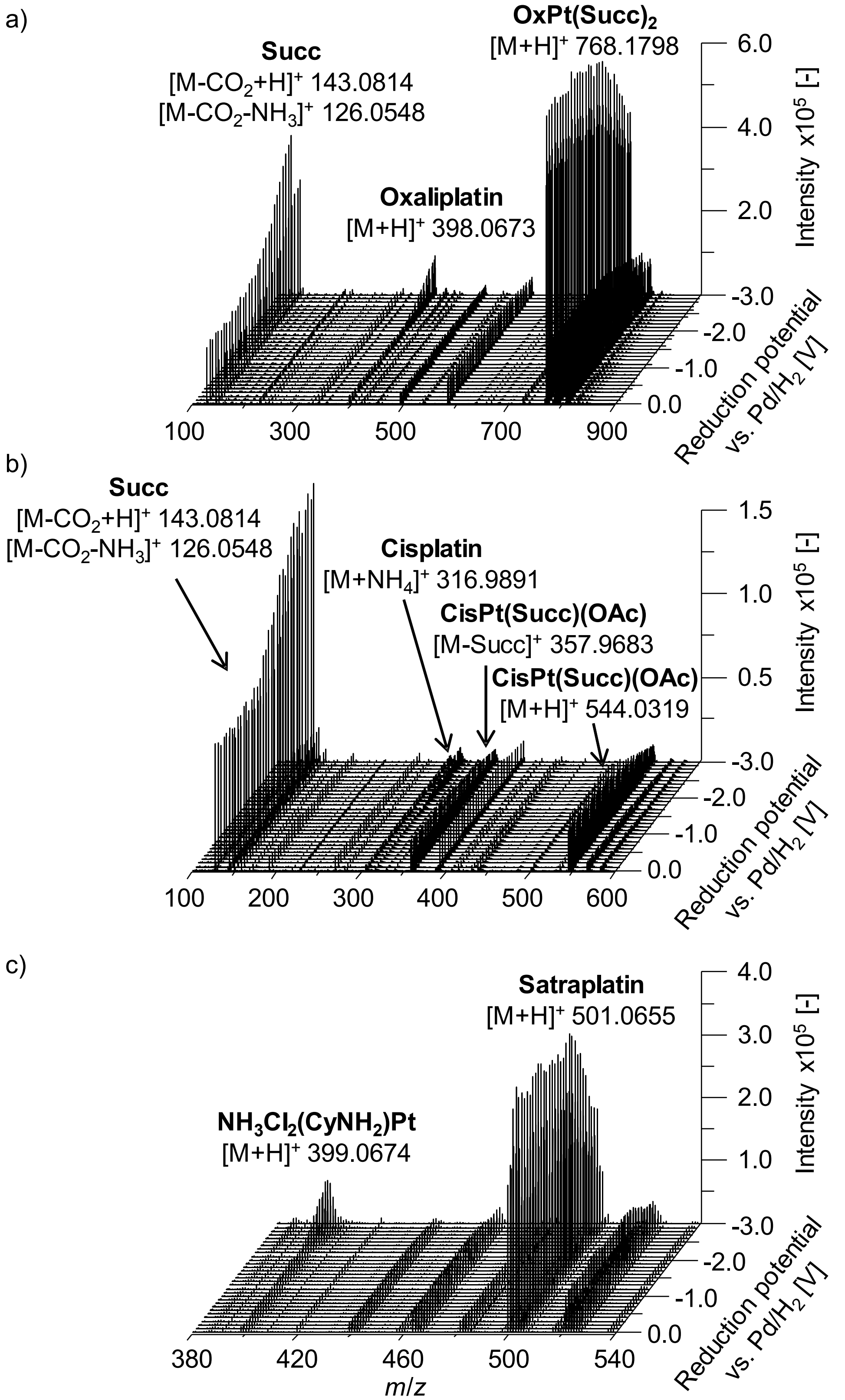

Fig. S1: Mass voltammograms of a) OxPt(Succ)$_2$, b) CisPt(Succ)(OAc) and c) satraplatin. A potential ramp from 0.0 to -3.0 V (vs. Pd/H2) was applied to the electrochemical cell.

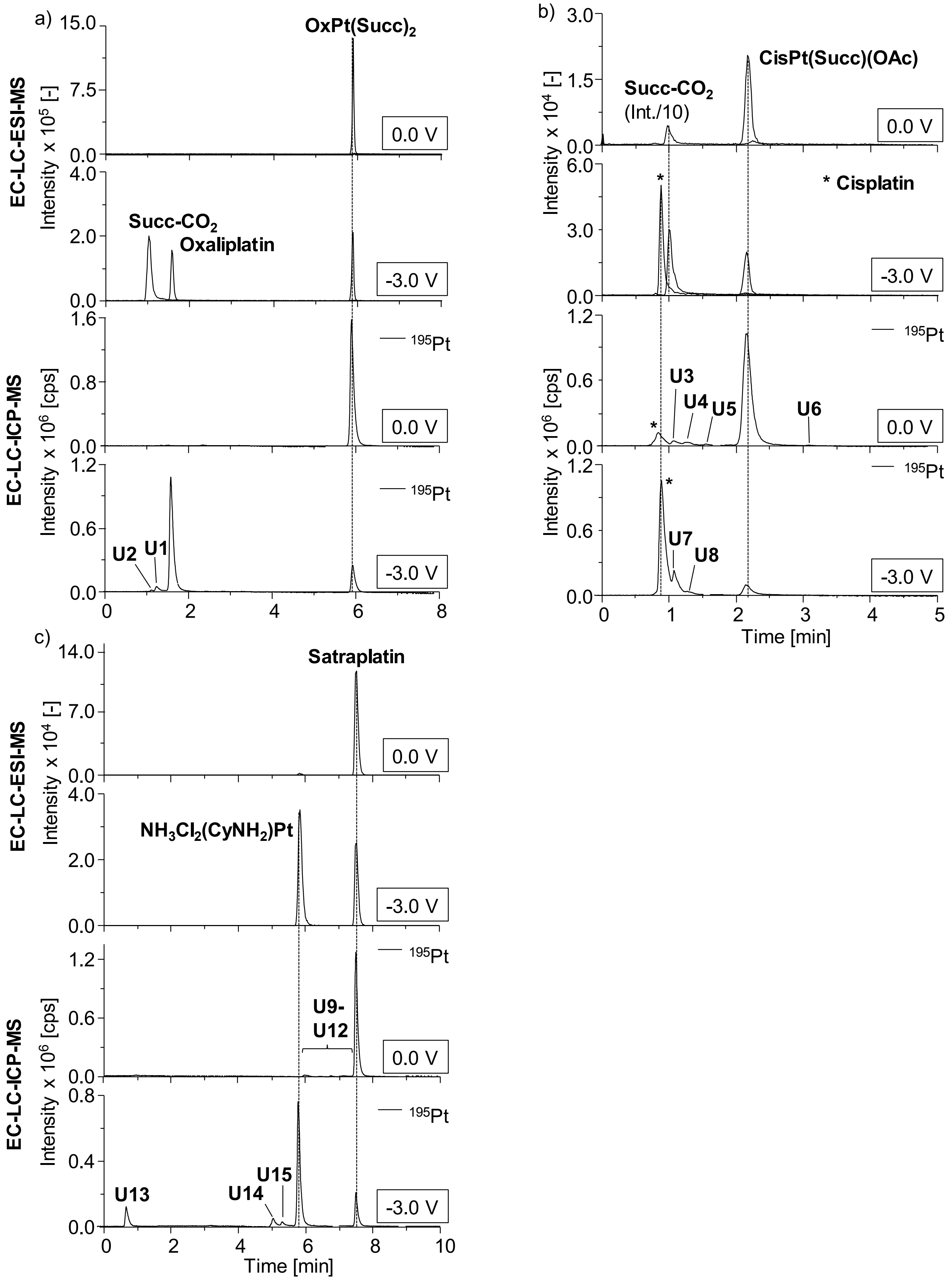

Fig. S2: Chromatographic separation of a) $OxPt(Succ)_2$, b) CisPt(Succ)(OAc) and c) satraplatin and their respective reduction products obtained by electrochemical conversion at a constant potential of -3.0 V vs. Pd/H_2. Detection was performed using ESI-MS and complementary ICP-MS.

Table S5: Overview of detected products obtained by electrochemical reduction of Pt(IV) complexes. Listed are the detected species with ESI-MS and/or ICP-MS, respectively, as well as their retention times, determined Pt concentration and the corresponding relative standard deviation [%]. In addition, the total recovery of Pt is shown.

Pt(IV) compound	potential vs. Pd/H_2 [V]	species	ESI-MS	ICP-MS	t_R [min]	$c(^{195}Pt)$ [µg/L]	RSD [%]	Pt recovery [%]
**OxPt(Succ)(OAc)**	**0.0**	OxPt(Succ)(OAc)	+	+	4.6	976	5.3	100
	**-3.0**	OxPt(Succ)(OAc)	+	+	4.6	274	38	90
		OxPt	+	+	1.6	578	3.8	
		Succ	+	-	1.0	-	-	
		U1	-	+	1.3	26.5	12	
**$OxPt(Succ)_2$**	**0.0**	$OxPt(Succ)_2$	+	+	6.0	1045	3.3	107
	**-3.0**	$OxPt(Succ)_2$	+	+	6.0	199	6.1	86
		OxPt	+	+	1.6	611	11	
		Succ	+	-	1.0	-	-	
		U1	-	+	1.3	26.0	2.2	
		U2	-	+	1.1	5.25	8.0	
**CisPt(Succ)(OAc)**	**0.0**	CisPt(Succ)(OAc)	+	+	2.1	795	0.2	97
		CisPt	+	+	0.7	90.5	1.1	
		U3	-	+	1.0	27.7	3.5	
		U4	-	+	1.2	27.0	4.8	
		U5	-	+	1.5	10.9	0.1	
		U6	-	+	3.1	6.94	5.7	
	**-3.0**	CisPt(Succ)(OAc)	+	+	2.1	119	12	84
		CisPt	+	+	0.7	580	5.8	
		Succ	+	-	1.0	-	-	
		U7	-	+	1.1	87.2	39	
		U8	-	+	1.3	33.6	10	
**satraplatin**	**0.0**	satraplatin	+	+	7.7	1007	4.9	106
		U9	-	+	6.2	10.4	53	
		U10	-	+	6.6	3.19	29	
		U11	-	+	6.9	6.90	3.7	
		U12	-	+	7.3	3.40	8.7	
	**-3.0**	satraplatin	+	+	7.7	195	57	92
		$NH_3Cl_2(CyNH_2)Pt$	+	+	6.1	573	4.7	
		U13	-	+	0.9	67.7	13	
		U14	-	+	5.4	34.9	14	
		U15	-	+	5.7	24.7	50	

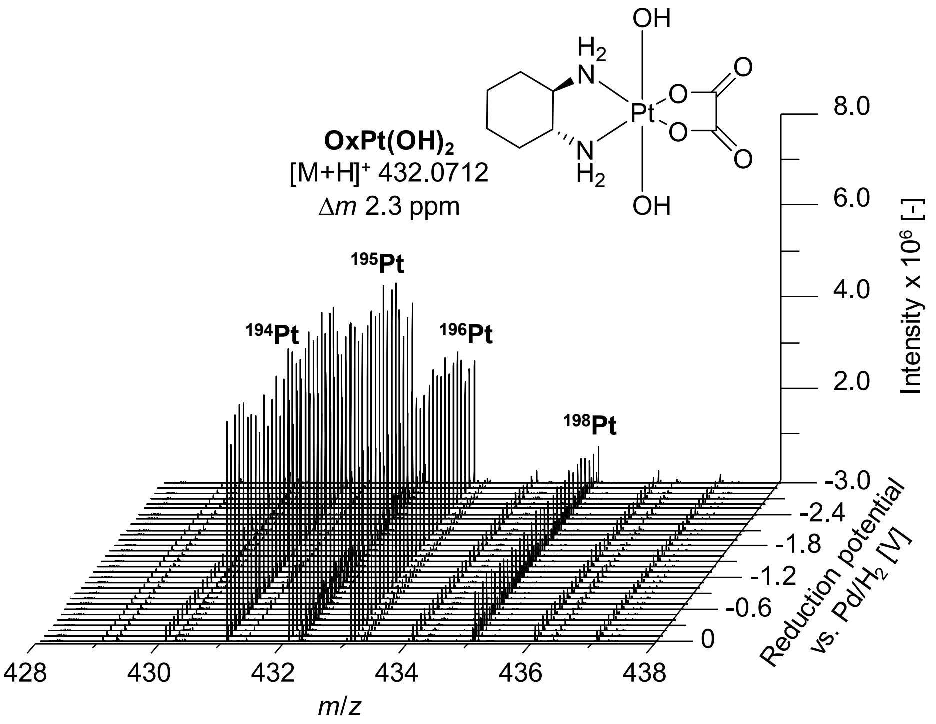

Fig. S3: Mass voltammograms of $OxPt(OH)_2$. A potential ramp from 0.0 to -3.0 V (vs. Pd/H_2) was applied to the electrochemical cell.
